# Mortality and morbidity of low-grade red blood cell transfusions in septic patients: a propensity score-matched observational study of a liberal transfusion strategy

**DOI:** 10.1186/s13613-020-00727-y

**Published:** 2020-08-08

**Authors:** Caroline Ulfsdotter Nilsson, Peter Bentzer, Linnéa E. Andersson, Sofia A. Björkman, Fredrik P. Hanssson, Thomas Kander

**Affiliations:** 1 Department of Intensive and Perioperative Care, Skåne University Hospital Lund, 221 85 Lund, Sweden; 2 Department of Clinical Sciences, Anaesthesiology and Intensive Care, Lund University, 221 85 Lund, Sweden; 3 Department of Anaesthesiology and Intensive Care, Helsingborg Hospital, 252 23 Helsingborg, Sweden; 4 Clinical Trial Consultants, Dag Hammarskjöldsväg 10b, 752 37 Uppsala, Sweden

**Keywords:** Red blood cell transfusion, Blood transfusion, Severe sepsis, Septic shock, Circulatory failure, Respiratory failure, Renal failure, Critical care

## Abstract

**Background:**

Red blood cell (RBC) transfusions are associated with risks including immunological reactions and volume overload. Current guidelines suggest a restrictive transfusion strategy in most patients with sepsis but based on previous randomized controlled trials and observational studies, there are still uncertainties about the safety in giving low-grade RBC transfusions to patients with sepsis.

**Methods:**

Critically ill patients with severe sepsis or septic shock admitted to a university hospital intensive care unit between 2007 and 2018 that received less or equal to 2 units of RBCs during the first 5 days of admission were propensity score matched to controls. Outcomes were 90- and 180-day mortality, highest acute kidney injury network (AKIN) score the first 10 days, days alive and free of organ support the first 28 days after admission to the intensive care unit and highest sequential organ failure assessment score (SOFA-max).

**Results:**

Of 9490 admissions, 1347 were diagnosed with severe sepsis or septic shock. Propensity-score matching resulted in two well-matched groups with 237 patients in each. The annual inclusion rate in both groups was similar. The median hemoglobin level before RBC transfusion was 95 g/L (interquartile range 88–104) and the majority of the patients were transfused in first 2 days of admission. Low-grade RBC transfusion was associated with increased 90- and 180-day mortality with an absolute risk increase for death 9.3% (95% confidence interval: 0.6–18%, *P* = 0.032) and 11% (95% confidence interval: 1.7–19%, *P* = 0.018), respectively. Low-grade RBC transfusion also correlated with increased kidney, circulatory and respiratory failure and higher SOFA-max score.

**Conclusions:**

Low-grade RBC transfusion during the first 5 days of admission was associated with increased mortality and morbidity in a liberal transfusion setting. The results support the current practice of a restrictive transfusion strategy in septic critically ill patients.

## Background

Critically ill patients with sepsis are frequently anemic. The underlying pathophysiology is multifactorial and includes production of inflammatory cytokines that increase hepcidin which reduces iron availability [1], dilution due to fluid therapy, and blood loss [2]. Red blood cell (RBC) transfusions to correct anemia can be life-saving but are also associated with a number of potential adverse effects which makes risk–benefit assessment challenging [3–5]. The adverse effects include infections, hemolytic reactions, transfusion-related acute lung injury (TRALI), pulmonary edema due to volume overload (transfusion-associated cardiac overload, TACO) and effects on the immune system with transfusion-related immunomodulation (TRIM) [6].

The clinical impact of the adverse effects on morbidity and mortality of transfusion of RBCs in sepsis has been investigated in randomized controlled trials (RCT) [7–9]. In the subgroup analyses of septic patients in the TRICC trail [7] and in the TRISS trail [8], a liberal RBC transfusion strategy (hemoglobin level > 90–100 g/L) did not confer a benefit as compared to a restrictive strategy (hemoglobin level > 70 g/L). However, in the TRICOP trial, performed in a cohort of septic oncology patients, a liberal transfusion strategy was associated with a lower 90-day mortality [9]. Furthermore, observational studies have also demonstrated positive effects of RBC transfusions. [10, 11].

Thus, these data are inconclusive and areas of uncertainty remain. In the RCTs, the time between admission and inclusion was 6 h or longer, which means that the effect of any RBC transfusion given early in the course of sepsis was not studied. Moreover, patients in the restrictive group received on average one unit of blood, meaning that the potential adverse effects of a low dose of RBC transfusion could not be assessed. Furthermore, a higher proportion of patients in the restrictive group discontinued the study which could have biased the results.

In an attempt to address some of these uncertainties, we propensity score-matched patients with severe sepsis or septic shock who received low-grade RBC transfusions any of the first 5 days after intensive care unit (ICU) admission to those who did not receive RBC transfusions and evaluated the effect on mortality and organ failure.

## Methods

### Data collection and study population

This study was approved by Swedish Ethical Review Authority in Lund, Sweden (registration numbers 2014/916 and 2018/866). All participants were offered an opt-out via advertisement in the local newspaper and the board waived the requirement for written informed consent. The manuscript was prepared according to the STROBE guidelines for observational studies [12].

All patients ≥ the age of 18 admitted to the 9-bed general ICU at Skåne University Hospital, Lund, Sweden between 2007 and 2018 with severe sepsis or septic shock according to the Sepsis-2 definition were eligible for inclusion [13]. For patients with multiple admissions with the diagnosis of severe sepsis or septic shock, only the first admission was included in the study. Day 0 started at admission and ended at 06:00. As described above, a condition with massive bleeding can affect outcome and patients who received high-grade RBC transfusion (> 670 ml [= more than 2 units]) during the first 5 days were, therefore, excluded. RBC transfusions were given at the discretion of the treating physician but in local guidelines, it was recommended to keep hemoglobin level above 100 g/L in critically ill patients with severe sepsis or septic shock.

Mortality data were extracted from the Swedish intensive care quality register PASIVA (Otimo Data AB, Kalmar, Sweden). Physiological and laboratory data and pre-existing conditions (age, gender, chronic obstructive pulmonary disease (COPD), renal failure, diabetes), outcome variables (except mortality) and fluid administration data were collected from raw data, i.e., from the electronic master chart system of the hospital (Melior, Cerner, N. Kansas City, MO, USA), or from the patient data management system at the ICU (Intellispace critical care and anaesthesia (ICCA), Philips, Amsterdam, the Netherlands).

### Outcome variables

Mortality was assessed at 90 and 180 days after ICU admission. Organ support was assessed by calculating days alive and free (DAF) of organ support for the first 28 days after admission to the ICU [14]. For patients who died in the ICU, we counted the days without the specified organ support before death. Organ support measures were vasopressors for circulatory failure, mechanical invasive ventilation for respiratory failure and renal replacement therapy (RRT) for kidney failure. To further assess organ failure, the maximum sequential organ failure assessment (SOFA) score during the first 28 days after admission was analyzed. Kidney failure was also evaluated according to the acute kidney injury network (AKIN) scoring system [15]. The maximal AKIN score the first 10 days after ICU admission was used for analysis.

### Statistics

Patients receiving low-grade RBC transfusion during the first 5 days of ICU admission were propensity score matched with non-transfused patients to adjust for differences in baseline variables associated with outcome. The propensity score was calculated with linear logistic regression using a one-to-many macro for SAS [Parsons 2004] with the covariates specified in Table 1. Physiological and laboratory variables used in the propensity score matching were collected within 90 min of admission to the ICU. Note that the hemoglobin value at admission was not included in the matching in the primary analysis. In a secondary sensitivity analysis, the median hemoglobin value day 0 was included in the matching. A greedy matching procedure in both the primary and secondary analyses matched treated to controls at a ratio of 1:1. In a first step, a match was sought with a propensity score that was identical to 8 decimal places to the treated patient. If no match was found, a match would be sought at 7 decimal places and so on. If no match was found at one decimal place, the patient receiving RBC transfusion was excluded from the study. A control could only be used once. A standardized difference of < 10% has previously been suggested to indicate negligible differences in the mean or prevalence of covariates between groups [16, 17].

**Table 1:** Patient demographics before and after propensity matching

	Unmatched groups		Standardized difference	*P* value	Propensity-matched groups		Standardized difference	*P* value
	Control *N* = 405	RBC^a^*N* = 459			Control *N* = 237	RBC *N* = 237		
Pre-existing conditions								
Age, mean (SD^b^)	64 (16)	65 (15)	0.047	0.490	65 (15)	65 (15)	0.010	0.914
Male gender, no (%)	233 (57)	234 (51)	0.131	0.054	130 (55)	138 (58)	0.068	0.460
Blood malignancy^c^, no (%)	16 (4.4)	42 (9.2)	0.187	0.007	12 (5.1)	14 (5.9)	0.037	0.687
COPD^d^, no (%)	42 (10) (0.305)	52 (11)	0.030	0.652	29 (12)	28 (12)	0.013	0.888
Cirrhosis, no (%)	17 (4.2)	12 (2.6)	0.087	0.198	11 (4.6)	8 (3.4)	0.065	0.483
Immunosuppression^e^, no (%)	30 (7.4)	54 (12)	0.148	0.031	18 (7.6)	19 (8.0)	0.016	0.864
Malignancy^f^, no (%)	43 (11)	73 (16)	0.156	0.023	31 (13)	33 (14)	0.025	0.789
Nosocomial infection^g^, no (%)	33 (8.1)	44 (9.6)	0.051	0.460	21 (8.9)	24 (10)	0.043	0.639
Airway infection, no (%)	112 (28)	116 (25)	0.054	0.428	63 (27)	63 (27	0	1.000
Surgery^h^, no (%)	64 (16)	99 (22)	0.148	0.031	40 (17)	48 (20)	0.087	0.346
GI^i^-bleeding, no (%)	0 (0)	6 (1.3)	0.163	0.021	0 (0)	0 (0)	0.000	1.000
DIC^j^, no (%)	25 (6.2)	33 (7.2)	0.041	0.552	19 (8.0)	14 (5.9)	0.083	0.368
I.C.^k^ volume effect, no (%)	2 (0.5)	4 (0.9)	0.046	0.505	2 (0.8)	2 (0.8)	0	1.000
Physiological and laboratory variables at admission^l^, mean (SD)								
Heart rate, mean (SD)	107 (23)	109 (24)	0.085	0.216	106 (23)	106 (25)	0.013	0.889
SBP^m^, (mmHg)	108 (30)	107 (29)	0.052	0.450	107 (30)	108 (31)	0.044	0.634
Lactate (mmol/L)	3.4 (3.2)	3.5 (2.7)	0.027	0.691	3.4 (3.2)	3.4 (2.7)	0	0.996
Norepinephrine (µg/min)	7.0 (12)	11.6 (20)	0.285	< 0.001	9.0 (13)	7.7 (11)	0.108	0.241
Temperature (°Celcius)	37.4 (1.5)	37.4 (1.4)	0.035	0.617	37.3 (1.5)	37.3 (1.4)	0.007	0.940
PaO_2_/FiO_2_ (kPa)	23 (15)	22 (16)	0.040	0.588	23 (15)	22 (15)	0.040	0.665
Leucocytes (× 10^9^/L)	16 (18)	14 (19)	0.093	0.197	16 (20)	15 (20)	0.038	0.683
Platelets (× 10^9^/L)	188 (130)	182 (135)	0.044	0.517	187 (129)	185 (120)	0.018	0.845
pH	7.13 (1.5)	7.31 (0.50)	0.261	<0.001	7.34 (0.12)	7.34 (0.11)	0.042	0.647
Bilirubin (µmol/L)	24 (35)	25 (46)	0.010	0.891	24 (33)	26 (50)	0.053	0.561
Creatinine (µmol/L)	172 (126)	180 (141)	0.063	0.360	181(130)	175 (133)	0.042	0.645
PT/INR^n^	1.58 (0.85)	1.62 (0.82)	0.040	0.550	1.58 (0.70)	1.59 (0.78)	0.005	0.956
APTT^o^ (sec)	42 (19)	45 (17)	0.178	0.009	43 (18)	42 (14)	0.050	0.586

^a^Low-grade red blood cell transfusion defined as < 670 ml any of the first 5 days

^b^Standard deviation

^c^Lymphoma, acute leukaemia or myeloma

^d^Chronic obstructive pulmonary disease

^e^Chronic steroid treatment correlative to ≥ 0.3 mg/kg prednisolone/day, radiation, or chemo therapy

^f^Cancer spread beyond the regional lymph nodes

^g^Infection that developed after ≥ 48 h in hospital or secondary to surgical or medical procedure

^h^Before admission to intensive care

^i^Gastro-intestinal

^j^Disseminated intravascular coagulopathy

^k^Intra-cranial

^l^First value within 90 min after admission except for “Norepinephrine” which is the mean dose the first 12 h

^m^Systolic blood pressure

^n^Prothrombin time

^o^Activated partial thromboplastin time

Sample size was based on the number of available patients during the study period. Variables were summarized using mean or median with standard deviation or range as distribution measurement. The propensity score matching was performed using SAS version 9.4 (SAS Institute Inc., Cary, NC, USA) prior to any comparison between the groups. Kaplan–Meier survival analysis was performed and is presented in graphs with corresponding stratified log-rank test. In accordance with the previous recommendations [18] comparisons between the groups after propensity score matching was performed with paired hypothesis testing using SPSS Statistics version 24 (SPSS Inc., Chicago, Ill., USA). A two-sided P value of less than 0.05 was considered to indicate statistical significance. Continuous variables are presented as median (interquartile range) and all categorical variables are presented as numbers (percentage).

## Results

### Study population and propensity score match

**Fig. 1:**
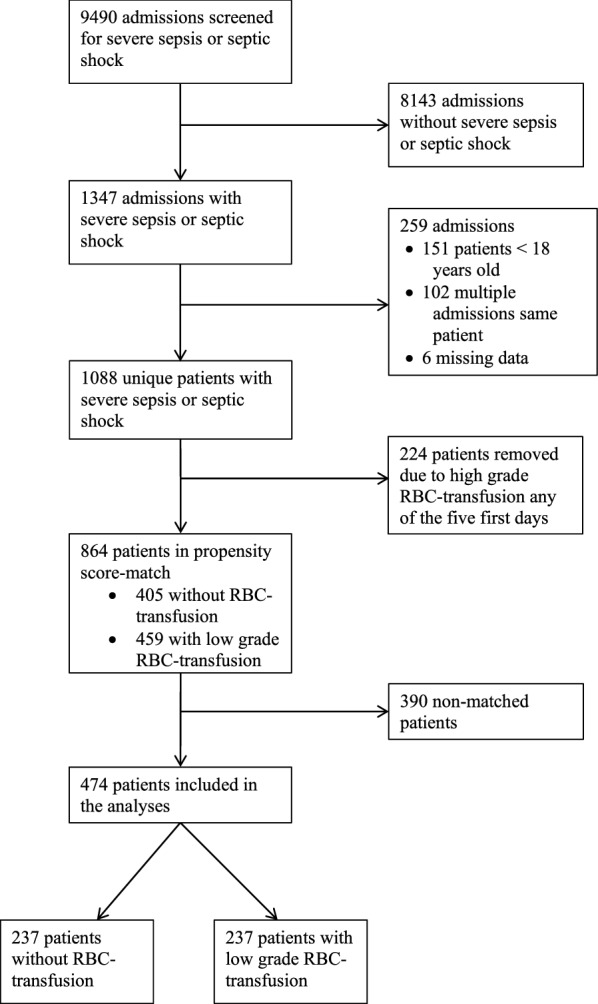
Consort diagram

Of 9490 ICU admissions 1347 were diagnosed with severe sepsis or septic shock. After elimination of multiple admissions, age < 18 and patients who received high-grade transfusion, 864 patients were included. The propensity score match of these patients resulted in 237 patients in the RBC group and 237 patients in the control group (Fig. 1). The rate of inclusion per year in respective group is illustrated in Additional file 1. Baseline values including comorbidity, special treatments, vital signs and laboratory results at admission in the unmatched and matched study population are summarized in Tables 1 and 2. Matching reduced standardized difference between the groups in baseline characteristics to less than 10% for all variables except norepinephrine. For baseline variables not included in the matching, differences between the groups were eliminated after the matching for all variables except for “Reason for admission, Gastric”, Table 2.

**Table 2:** Unmatched baseline characteristics

	Unmatched groups			Propensity-matched groups		
	Controls, *n* = 405	RBC^a^, *n* = 459	*P*-value^b^	Controls, *n* = 237	RBC, *n* = 237	*P* value^c^
SAPS 3^d^, median (IQR^e^)	66 (58–77)	71 (62–81)	< 0.001	69 (60–80)	69 (59–79)	N.S.
First SOFA^f^	8 (5–10)	9 (6–11)	< 0.001	8 (5–11)	8 (5–10)	N.S.
Days before ICU^g^	0 (0–2)	1 (0–5)	< 0.001	0 (0–2)	1 (0–3)	N.S.
Reasons for admission^h^, *n* (%)						
CNS^i^	91 (22)	92 (20)	N.S.	58 (25)	57 (24)	N.S.
Hematologic	39 (9.6)	64 (14)	N.S.	26 (11)	27 (11)	N.S.
Gastric	88 (22)	148 (32)	< 0.001	51 (21)	74 (31)	0.02
Metabolic	82 (20)	95 (21)	N.S.	53 (22)	51 (21)	N.S.
Respiratory	188 (146)	239 (52)	N.S	111 (47)	130 (55)	N.S.
Cardiovascular	67 (17)	75 (16)	N.S.	35 (15)	41 (17)	N.S.
Hepatic	29 (7.2)	43 (9.4)	N.S.	22 (9.3)	22 (9.3)	N.S.
Renal	138 (34)	160 (35)	N.S.	89 (38)	93 (39)	N.S.
Other	42 (10)	34 (7.4)	N.S.	24 (10)	26 (11)	N.S.
Arrival route *n* (%)						
Emergency department	115 (28)	93 (20)	0.007	64 (27)	54 (23)	N.S.
General ward	199 (49)	232 (51)	N.S.	128 (54)	120 (51)	N.S.
Intermediate care	7 (1.7)	19 (4.1)	0.046	3 (1.3)	11 (4.6)	N.S.
Operation	30 (7.4)	37 (8.0)	N.S.	21 (8.9)	22 (9.2)	N.S.
Other ICU	39 (9.6)	48 (10)	N.S.	20 (8.4)	29 (12)	N.S.
Other arrival route	15 (3.7)	30 (6.5)	N.S.	1 (0.42)	1 (0.42)	N.S.

^a^Red blood cell transfusion

^b^Mann–Whitney or Chi-2 test

^c^Wilcoxon rank sum or McNemar´s test

^d^Simplified acute physiology score 3

^e^Interquartile range

^f^Sequential organ failure assessment

^g^Days on hospital before admission to the intensive care unit

^h^Patients may have more than one reason for admission

^i^Central nervous system

**Fig. 2:**
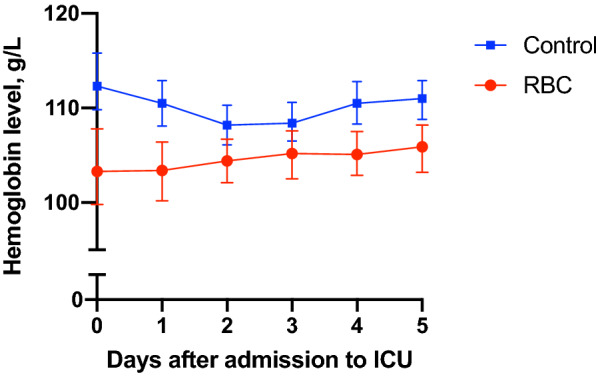
Median hemoglobin level with interquartile range. RBC = group with patients who received red blood cell transfusion any of the first 5 days

**Fig. 3:**
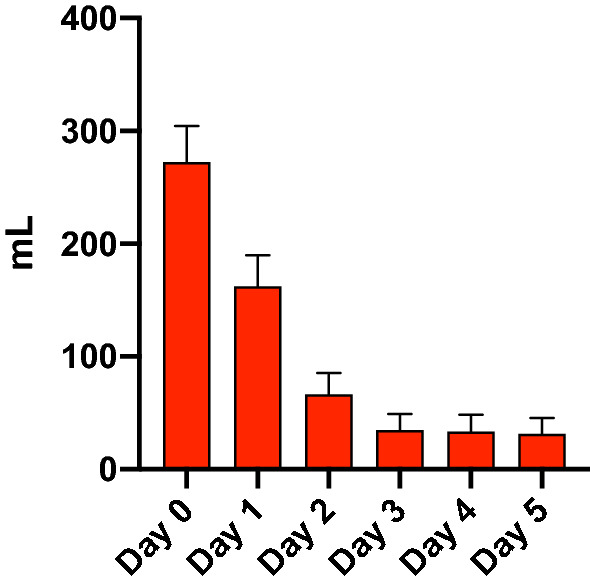
Mean red blood cell transfusion per day with 95% confidence interval in the RBC group. RBC = group

All RBC transfusions were leucoreduced. Median hemoglobin level in the RBC group immediately before RBC transfusion was 95 g/L (88–104). Immediately after the RBC transfusion, the median hemoglobin level was 101 g/L (93–109). The median hemoglobin level on day 0 was 103 g/L (95–108) for the RBC group and 117 g/L (104–131) for the control group (*P* < 0.001). Daily hemoglobin levels during the first 5 days of ICU admission in both groups are shown in Fig. 2. The median volume of RBC transfusion in the RBC group the first five days after admission was 177 ml/day (110–291). The majority of patients were transfused during the first 2 days of admission (Fig. 3).

### Outcomes

**Fig. 4:**
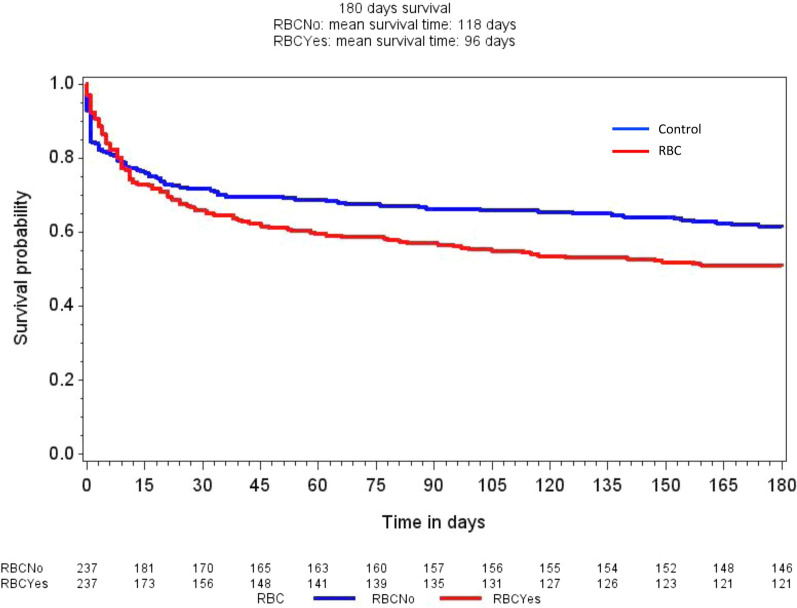
Kaplan–Meier curves of 180-day survival in the control group (blue line) and the RBC group (red line) (*P* = 0.046, stratified log-rank test). RBC = group with patients who received red blood cell transfusion any of the first 5 days

Detailed results of the outcome measures are presented in Table 3. In summary, mortality for patients in the RBC group was higher at 90 and 180 days compared to patients in the control group (Table 3 and Fig. 4). The absolute risk increase for death at 180 days for patients in the RBC group was 11% (95% CI 1.7–19%); (*P* = 0.018). The AKIN max was higher in the RBC group and patients in the RBC group were more likely to receive RRT, compared to patients in the control group. There was no difference in DAF of RRT between the groups. DAF of vasopressors and mechanical ventilation was lower in the RBC group than in the control group, indicating more pronounced circulatory and respiratory failure in the RBC group. Patients in the RBC group demonstrated a higher median SOFA-max score than patients in the control group.

**Table 3:** Main outcome variables

Outcome	Propensity-matched groups		Relative risk (95% CI^a^)	Absolute risk increase (95% CI)	*P*^b^
	Control *n* = 237	RBC^c^*n* = 237			
90-day mortality	80 (34)	102 (43)	1.28 (1.01–1.61)	9.3% (0.6–18%)	0.032
180-day mortality	91 (38)	116 (49)	1.27 (1.04–1.57)	11% (1.7–19%)	0.018
RRT^d^	21 (8.9)	48 (20)	2.29 (1.41–3.69)	11% (5.1–18%)	0.001
AKIN	0 (0–2)	0 (0–3)			0.001
DAF^e^ of RRT	28 (16–28)	28 (9–28)			0.29
DAF of vasopressors	26 (13–27)	23 (7–26)			0.009
DAF of mechanical ventilation	26 (12–28)	21 (4–27)			0.002
SOFA max^f^	9 (7–12)	11 (8–13)			< 0.001

Data are presented as number (%) or median (interquartile range)

^a^Confidence interval

^b^Wilcoxon rank sum or McNemar´s test

^c^Low-grade red blood cell transfusion defined as < 670 ml any of the first 5 days

^d^Renal Replacement Therapy

^e^Days Alive and Free

^f^Sequential Organ Failure Assessment score the first 10 days after admission

### Fluids and RBC transfusion

During the first 5 days of ICU stay, there was no difference in crystalloid or colloid administration or total fluid balance between the two groups (Table 4). Patients in the RBC group received more total fluids and produced more urine the first 5 days compared to the control group.

**Table 4:** Fluid therapy, first 5 days

Fluids per day	Propensity score-matched groups				*P*^b^
	Control, *n* = 237		RBC^a^, *n* = 237		
	Median	IQR^c^	Mediann	IQR	
Colloids^d^ (ml)	342	100–688	357	206–621	0.939
Crystalloids^e^ (ml)	1200	482–2547	1120	500–2015	0.082
Fluids in, total^f^ (ml)	3495	2301–4557	3920	3127–4963	< 0.001
Urine output (ml)	1916	429–2772	2295	906–3134	0.005
Total fluid balance^g^ (ml)	544	0–2066	645	− 100 to 1568	0.765
RBC transfusion (ml)	0	0–0	177	110–291	< 0.001

For patients with ICU stay < 5 days, the mean per day was calculated for the length of stay

^a^Low-grade red blood cell transfusion defined as < 670 ml (< 2 units) any of the first 5 days

^b^Wilcoxon rank sum test

^c^Interquartile range

^d^Defined as albumin (200 mg/ml), albumin (5 mg/ml), dextran 70 (60 mg/ml) and hydroxyethyl starch (200/0.5 and 130/0.4)

^e^Crystalloids represents the sum of NaCl 9 mg/ml and Ringer´s Acetate

^f^Fluids in, total represents the sum of all enteral and parenteral administered fluids but not RBC transfusions

^g^Insensible perspiration and RBC transfusions not included

### Sensitivity analysis

**Fig. 5:**
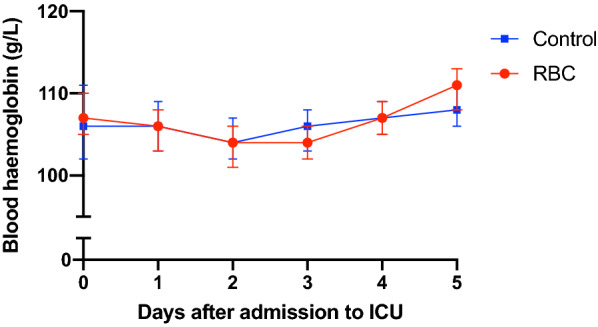
Median hemoglobin level with interquartile range in the sensitivity analysis that included hemoglobin level day 0 in the matching. RBC = group with patients who received red blood cell transfusion any of the first 5 days

The sensitivity analysis was performed to investigate any effects of hemoglobin level at admission on the outcomes. This was done by including the median hemoglobin level the admission day in the matching protocol. In this analysis, the starting point was the same original population of 864 patients with severe sepsis or septic shock as in the main analysis (Fig. 1). The propensity score matching resulted in 116 patients in the control group and 116 patients in the RBC group. The matching was good with < 10% standardized difference in all variables except 3 (systolic blood pressure, platelets and creatinine) (Additional file 2). Median hemoglobin level in the RBC group immediately before RBC transfusion was 95 g/L (88–103). The median hemoglobin level day 0 was 107 g/L (105–110) for the RBC group and 106 g/L (102–111) for the control group. For hemoglobin levels the first 5 days in both groups please see Fig. 5. The differences between groups were essentially unchanged compared to the primary analyses (Additional file 3).

## Discussion

In the present study, low-grade leukoreduced RBC transfusion in critically ill septic patients was associated with increased mortality, increased kidney, circulatory and respiratory failure as well as higher SOFA-max score.

With the propensity score matching, we aimed to create the RBC and the control groups as similar as possible with respect to severity of illness at ICU admission. Because a low hemoglobin at admission may be a result of an underlying condition which may influence outcome independent of RBC transfusions, we performed a sensitivity analysis in which hemoglobin concentration at admission was included in the propensity score matching. Although the results of this analysis are slightly less robust due to the lower number of matched patients and hence lower power, the results were very similar. Further, pre-matching differences between the groups in baseline variables not included in the propensity score matching were erased after the matching for all variables except for “Gastric reason for admission” (Table 2). Taken together, this supports the robustness in the propensity score matching and in the findings in the main analysis.

The observed hemoglobin level of 95 g/L before transfusion may appear high given that current guidelines suggest a transfusion trigger of 70 g/L in the absence of ongoing ischemia. As mentioned above, the present study was performed in an institution with a liberal tradition of RBC transfusions and before the publication of the TRISS trial a transfusion trigger of 100 g/L was accepted in hemodynamically unstable patients with sepsis. After 2014, a more restrictive approach was implemented and the observed hemoglobin level before transfusion, therefore, reflects this change of practice.

In contrast to our results, two randomized trials comparing liberal (hemoglobin level goal > 90–100 g/L) vs restrictive (> 70 g/L) transfusion strategy in critically ill patients with sepsis or septic shock did not detect a difference in mortality or need for vasopressors, mechanical ventilation or RRT between the treatment strategies [7, 8]. It should be noted that previous RCTs on different hemoglobin thresholds for RBC transfusions did not study the possible negative effects of RBC transfusions but rather the effects of different thresholds for RBC transfusions [7–9]. This means that also patients in the low threshold group received a significant number of RBC units. In contrary, the present study included a control group with patients who did not receive any RBC transfusions at all during the study period. Thus, when evaluating if RBC transfusions are harmful, independent of hemoglobin levels within a safe interval, observational studies like the present may have some benefits. Our result demonstrated that the majority of RBCs were administered within the first days after ICU admission. Interestingly, the largest RCT on transfusion thresholds in septic shock to date, the TRISS trial, included patients on average 22 h after admission and could not demonstrate a difference between a low and high transfusion trigger [8]. This raises the possibility that the effect of transfusions on outcome could be time dependent, in the sense that the early RBC transfusions given to septic patients might be the transfusions with highest risk.

As mentioned above, previous observational studies on the effect of RBC transfusions in sepsis have reported a decrease in mortality [10, 11]. In contrast to our study, these studies did not exclude patients receiving massive transfusions. Because massive transfusions indicate active bleeding, a condition in which the risk–benefit ratio may favor transfusion, it is possible that this difference in study design may explain the difference in results. Moreover, the average transfused patients in both of these studies were transfused at hemoglobin of 75–80 g/L which could contribute to the difference in results.

What is the potential pathophysiological mechanism of the observed increased mortality and morbidity after RBC low-grade transfusions in our study? As mentioned above, known adverse effects of RBC transfusion include TACO, TRALI and TRIM, all of which may cause negative effects in many organs [6]. Although patients in the RBC group received more fluids the first 5 days, the fluid balance was not different between the groups, which would indicate that fluid overload (TACO) was not the reason for the differences in outcome. The incidence of TRALI is previously estimated to be about one case per 12 000 transfusions [19]. Given that TRALI most commonly occurs after plasma transfusion and that no episodes of TRALI presentation were reported for included patients we believe that it is unlikely to the main cause of the differences between the groups even if underreported. TRIM represents an interaction of a multitude of immunomodulatory mediators in the RBC transfusion with the immune system, leading to both proinflammatory and immunosuppressive effects [20]. In the setting of sepsis, such effects may be deleterious and represents a potential mechanism by which RBCs may adversely affect outcome.

## Limitations

We recognize the limitations in the present study due to its retrospective nature. Although baseline values were carefully adjusted for severity of illness, the presence of undetected factors of relevance for outcome such as differences in comorbidities cannot be ruled out. Further, the study was done at a single department which limits the external validity. The risk–benefit ratio for transfusions is likely to be dependent on the Hb level at which RBC are transfused. Thus, it is important to emphasize that the results may only be valid for the transfusion level observed in our cohort and may not be generalized to other transfusion triggers.

Although there is evidence for the safety of a restrictive transfusion strategy in sepsis, we still lack knowledge of safety in certain sub-populations such as septic patients with myocardial ischemia, severe hypoxemia or acute hemorrhage. Furthermore, the potentially harmful effects of RBC transfusion on morbidity and long-term mortality warrants further evaluation including, e.g., individualized therapy and methods of preventing or treating anemia without RBC transfusion.

## Conclusion

In conclusion, this propensity score-matched study of patients with severe sepsis and septic shock with low-grade or no RBC transfusion indicates increased morbidity and long-term mortality of RBC transfusion in a liberal transfusion setting. These findings support the current Surviving sepsis campaign guidelines of restrictive transfusion strategy [21].

## Supplementary information

**Additional file 1:** Number of inclusions per year in the control and RBC group.

**Additional file 2:** Table of patient demographics before and after propensity matching, *sensitivity analysis*.

**Additional file 3:** Table of main outcome variables, *sensitivity analysis.*

## Data Availability

The datasets used and/or analyzed in the current study are available from the corresponding author on reasonable request.

## Abbreviations

AKIN: Acute kidney injury network
DAF: Days alive and free
ICU: Intensive care unit
RBC: Red blood cell
RCT: Randomized controlled trials
RRT: Renal replacement therapy
TACO: Transfusion-associated cardiac overload
TRALI: Transfusion-related acute lung injury
TRIM: Transfusion-related immunomodulation
