# Supplementary figures and images for: Mortality and morbidity of low-grade red blood cell transfusions in septic patients: a propensity score-matched observational study of a liberal transfusion strategy
Source: Ann Intensive Care. 2020 Aug 8;10:111. doi: 10.1186/s13613-020-00727-y (PMC7415067; doi:10.1186/s13613-020-00727-y)

## Inclusions per year

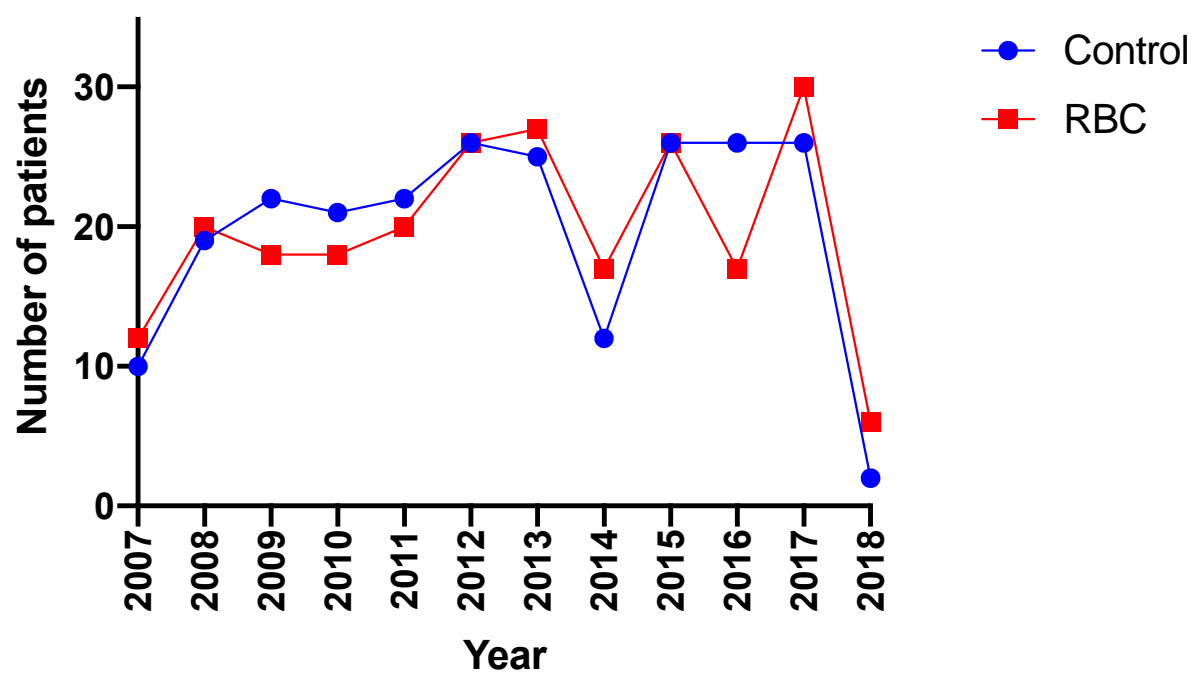

Supplement: Supplementary file 1 — Additional file 1: Number of inclusions per year in the control and RBC group. [file 13613_2020_727_MOESM1_ESM.pdf]
