# Supplementary material for: Mortality and morbidity of low-grade red blood cell transfusions in septic patients: a propensity score-matched observational study of a liberal transfusion strategy
Source: Ann Intensive Care. 2020 Aug 8;10:111. doi: 10.1186/s13613-020-00727-y (PMC7415067; doi:10.1186/s13613-020-00727-y)
Supplement: Supplementary file 2 — Additional file 2: Table of patient demographics before and after propensity matching, sensitivity analysis. [file 13613_2020_727_MOESM2_ESM.docx]

## Additional file 2. Patient demographics before and after propensity matching. Sensitivity analysis.

|  | **Unmatched groups** | **Standardized difference** | **P-value** | **Propensity-matched groups** | **Standardized difference** | **P-value** |
| --- | --- | --- | --- | --- | --- | --- |

|  | **Control**  **N= 405** | **RBC^[[1]](#endnote-1)^**  **N= 459** | |  |  | **Control**  **N= 116** | **RBC**  **N= 116** |  |  |
| --- | --- | --- | --- | --- | --- | --- | --- | --- | --- |
| **Pre-existing conditions** | | | | | | | | | |
| **Age, mean (SD^[[2]](#endnote-2)^)** | 64 (16) | | 65 (15) | 0.047 | 0.490 | 66 (15) | 67 (14) | 0.076 | 0.5622 |
| **Male gender, no (%)** | 233 (57) | | 234 (51) | 0.132 | 0.054 | 69 (60) | 67 (58) | 0.035 | 0.7909 |
| **Blood malignancy^[[3]](#endnote-3)^, no (%)** | 16 (4.4) | | 42 (9.2) | 0.188 | 0.007 | 4 (3.4) | 5 (4.3) | 0.045 | 0.7352 |
| **COPD^[[4]](#endnote-4)^, no (%)** | 42 (10) (0.305) | | 52 (11) | 0.031 | 0.652 | 14 (12) | 12 (10) | 0.055 | 0.6788 |
| **Cirrhosis, no (%)** | 17 (4.2)) | | 12 (2.6) | 0.087 | 0.198 | 5 (4.3) | 5 (4.3) | 0 | 1 |
| **Immunosuppression^[[5]](#endnote-5)^, no (%)** | 30 (7.4) | | 54 (12) | 0.148 | 0.031 | 11 (9.5) | 12 (10) | 0.029 | 0.827 |
| **Malignancy^[[6]](#endnote-6)^, no (%)** | 43 (11) | | 73 (16) | 0.156 | 0.023 | 17 (15) | 17 (15 | 0 | 1 |
| **Nosocomial infection^[[7]](#endnote-7)^, no (%)** | 33 (8.1) | | 44 (9.6) | 0.051 | 0.460 | 5 (4.3) | 4 (3.4) | 0.045 | 0.735 |
| **Airway infection, no (%)** | 112 (28) | | 116 (25) | 0.054 | 0.428 | 36 (31) | 35 (30) | 0.019 | 0.887 |
| **Surgery^[[8]](#endnote-8)^, no (%)** | 64 (16) | | 99 (22) | 0.148 | 0.031 | 19 (16) | 16 (14) | 0.072 | 0.584 |
| **GI^[[9]](#endnote-9)^-bleeding, no (%)** | 0 (0) | | 6 (1.3) | 0.163 | 0.021 | 0 (0) | 0 (0) | 0 | <0.001 |
| **DIC^[[10]](#endnote-10)^, no (%)** | 25 (6.2) | | 33 (7.2) | 0.041 | 0.552 | 9 (8.0) | 11 (9.5) | 0.061 | 0.641 |
| **I.C.^[[11]](#endnote-11)^ volume effect, no (%)** | 2 (0.5) | | 4 (0.9) | 0.046 | 0.505 | 1 (0.9) | 1 (0.9) | 0 | 1 |
| **Physiological and laboratory variables at admission^[[12]](#endnote-12)^, mean (SD )** | | | | | | | | | |
| **Heart rate, mean (SD )** | 107 (23) | | 109 (24) | 0.096 | 0.162 | 105 (21) | 105 (26) | 0.022 | 0.866 |
| **SBP^[[13]](#endnote-13)^, (mmHg)** | 108 (30) | | 107 (29) | 0.081 | 0.237 | 102 (27) | 98 (25) | 0.131 | 0.319 |
| **Lactate (mmol/L)** | 3.4 (3.2) | | 3.5 (2.7) | 0.015 | 0.821 | 3.5 (3.0) | 3.7 (2.6) | 0.081 | 0.538 |
| **Norepinephrine (µg/min)** | 7.0 (12) | | 11.6 (20) | 0.235 | 0.013 | 8.3 (12) | 7.5 (10) | 0.029 | 0.828 |
| **Temperature (°Celcius)** | 37.4 (1.5) | | 37.4 (1.4) | 0.035 | 0.617 | 37.3 (1.4) | 37.1 (1.3) | 0.097 | 0.461 |
| **PaO_2_/FiO_2_ (kPa)** | 23 (15) | | 22 (16) | 0.040 | 0.588 | 24 (15) | 24 (15) | 0.003 | 0.979 |
| **Leucocytes (x 10^9^/L)** | 16 (18) | | 14 (19) | 0.093 | 0.197 | 15 (00) | 15 (15) | 0.003 | 0.979 |
| **Platelets (x 10^9^/L)** | 188 (130) | | 182 (135) | 0.044 | 0.517 | 185 (122) | 172 (103) | 0.110 | 0.405 |
| **pH** | 7.13 (1.5) | | 7.31 (0.50) | 0.015 | 0.827 | 7.33 (0.12) | 7.34 (0.11) | 0.012 | 0.926 |
| **Bilirubin (µmol/L)** | 24 (35) | | 25 (46) | 0.010 | 0.891 | 25 (38) | 29 (59) | 0.081 | 0.539 |
| **Creatinine (µmol/L)** | 172 (126) | | 180 (141) | 0.043 | 0.535 | 195(130) | 181 (125) | 0.107 | 0.414 |
| **PT/INR^[[14]](#endnote-14)^** | 1.58 (0.85) | | 1.62 (0.82) | 0.003 | 0.967 | 1.66 (0.86) | 1.72 (1.0) | 0.061 | 0.645 |
| **APTT^[[15]](#endnote-15)^ (sec)** | 42 (19) | | 45 (17) | 0.135 | 0.049 | 42 (16) | 42 (12) | 0.043 | 0.741 |
| **Hb (g/L)^[[16]](#endnote-16)^** | 114 (19) | | 103 (14) | 0.682 | <0.001 | 108 (16) | 107 (21) | 0.088 | 0.504 |

1. Low grade red blood cell transfusion defined as <670 ml any of the first 5 days [↑](#endnote-ref-1)
2. Standard deviation [↑](#endnote-ref-2)
3. Lymphoma, acute leukaemia or myeloma [↑](#endnote-ref-3)
4. Chronic obstructive pulmonary disease [↑](#endnote-ref-4)
5. Chronic steroid treatment correlative to ≥ 0.3 mg/kg prednisolone/day, radiation, or chemo therapy [↑](#endnote-ref-5)
6. Cancer spread beyond the regional lymph nodes [↑](#endnote-ref-6)
7. Infection that developed after ≥ 48 hours in hospital or secondary to surgical or medical procedure [↑](#endnote-ref-7)
8. Before admission to intensive care [↑](#endnote-ref-8)
9. Gastro-intestinal [↑](#endnote-ref-9)
10. Disseminated intravascular coagulopathy [↑](#endnote-ref-10)
11. Intra-cranial [↑](#endnote-ref-11)
12. First value within 90 min after admission except for “Norepinephrine” which is the mean dose the first 12 hours [↑](#endnote-ref-12)
13. Systolic blood pressure [↑](#endnote-ref-13)
14. Prothrombin time [↑](#endnote-ref-14)
15. Activated partial thromboplastin time [↑](#endnote-ref-15)
16. Median hemoglobin level Day 0 [↑](#endnote-ref-16)
