# Supplementary material for: Mortality and morbidity of low-grade red blood cell transfusions in septic patients: a propensity score-matched observational study of a liberal transfusion strategy
Source: Ann Intensive Care. 2020 Aug 8;10:111. doi: 10.1186/s13613-020-00727-y (PMC7415067; doi:10.1186/s13613-020-00727-y)
Supplement: Supplementary file 3 — Additional file 3: Table of main outcome variables, sensitivity analysis. [file 13613_2020_727_MOESM3_ESM.docx]

## Additional file 3. Main outcome variables. Sensitivity analysis.

|  | **Propensity-matched groups** | | **Relative risk (95% CI^[[1]](#endnote-1)^)** | **Absolute risk increase (95% CI)** | **P^[[2]](#endnote-2)^** | |
| --- | --- | --- | --- | --- | --- | --- |
| **Outcome** | **Control**  **n= 116** | **RBC^[[3]](#endnote-3)^**  **n= 116** |  |  | |  |
| 90-day mortality | 41 (35) | 54 (47) | 1.3 (0.96 to 1.8) | 11% (-1.4 to 24%) | | 0.10 |
| 180-day mortality | 45 (38) | 61 (53) | 1.4 (1.02 to 1.8) | 14% (1.1 to 26%)) | | 0.04 |
| RRT^[[4]](#endnote-4)^ | 11 (9.5) | 29 (25) | 2.6 (1.4 to 5.0) | 16% (6.0 to 15%) | | 0.005 |
| AKIN max^[[5]](#endnote-5)^ | 0 (0-3) | 0.5 (0-3) |  |  | | 0.003 |
| DAF^[[6]](#endnote-6)^ of RRT | 28 (12-28) | 26 (11-28) |  |  | | 0.66 |
| DAF of vasopressors | 25 (10-26) | 22 (8-25) |  |  | | 0.11 |
| DAF of mechanical ventilation | 25 (8-28) | 21 (4-27) |  |  | | 0.06 |
| SOFA max^[[7]](#endnote-7)^ | 10 (8-13) | 12 (9-14) |  |  | | 0.002 |

Data are presented as median number (%) or (interquartile range)

1. Confidence interval [↑](#endnote-ref-1)
2. Wilcoxon rang sum or McNemar´s test [↑](#endnote-ref-2)
3. Low grade red blood cell transfusion defined as <670 ml any of the first 5 days [↑](#endnote-ref-3)
4. Renal Replacement Therapy [↑](#endnote-ref-4)
5. Maximal Acute Kidney Injury Network classification score the first 10 days after admission [↑](#endnote-ref-5)
6. Days Alive and Free [↑](#endnote-ref-6)
7. Sequential Organ Failure Assesment score the first 10 days after admission [↑](#endnote-ref-7)
